# ECDC’s latest publications

**DOI:** 10.2807/1560-7917.ES.2016.21.40.30362

**Published:** 2016-10-06

**Authors:** Eurosurveillance editorial team

**Affiliations:** 1 European Centre for Disease Prevention and Control (ECDC), Stockholm, Sweden

**Keywords:** public health

**Rapid risk assessments**
Date of publication: 9 September 2016
Crimean–Congo haemorrhagic fever in Spain

http://ecdc.europa.eu/en/publications/Publications/crimean-congo-haemorrhagic-fever-spain-risk-assessment.pdf

Date of publication: 6 September 2016
Multi-country outbreak of *Salmonella* Enteritidis phagetype 8, MLVA type 2-9-7-3-2 infections
http://ecdc.europa.eu/en/publications/Publications/29-08-2016-RRA-Salmonella%20Enteritidis-United%20Kingdom,%20Netherlands.pdf

**Surveillance reports**
Date of publication: 1 September 2016
Measles and rubella monitoring, July 2016

http://ecdc.europa.eu/en/publications/Publications/measles-rubella-monitoring-july-2016.pdf

Date of publication: every Friday
ECDC communicable disease threats report

http://ecdc.europa.eu/en/publications/surveillance_reports/Communicable-Disease-Threats-Report/Pages/default.aspx

